# Exploring topical atorvastatin hyalurosomal gel as an adjuvant for reducing systemic corticosteroid dosage: a randomized clinical trial in severe oral lichen planus patients

**DOI:** 10.1007/s13346-025-01956-z

**Published:** 2025-09-05

**Authors:** Mahitab Elsayed, Aya Essawy, Radwa M. Ismail, Yasmine Gamil, Mohamed G. Hamed, Dalia Elsabaawy, Eman Abdelhakeem, Doaa Hegazy, Radwa M. A. Abd-Elal

**Affiliations:** 1 Clinical Pharmacy Department, Faculty of Pharmacy, Modern University for Technology & Information, Cairo, Egypt; 2 Oral Medicine, Periodontology and Oral Diagnosis, College of Oral and Dental Surgery, Misr University for Science and Technology, Cairo, Egypt; 3 Department of Oral Medicine and Periodontology, Faculty of Dentistry, Modern University for Technology & Information, Cairo, Egypt; 4 Otolaryngology, Faculty of Medicine, Helwan University, Cairo, Egypt; 5 Clinical Pharmacy Department, Faculty of Pharmacy, Menofia University, Cairo, Egypt; 6 Department of Pharmaceutics and Industrial Pharmacy, Faculty of Pharmacy, Cairo University, Cairo, Egypt; 7 Pharmaceutics and Drug Manufacturing Department, Faculty of Pharmacy, Modern University for Technology and Information (MTI), Cairo, 11571 Egypt

**Keywords:** Atorvastatin, Clinical study, Hyalurosomes, I-optimal mixture design, OLP

## Abstract

Oral lichen planus (OLP) is a chronic inflammatory disorder with limited topical treatment options and long-term corticosteroid dependency. This study investigates a novel atorvastatin-loaded hyalurosomal gel (ATV-Hyalugel) as a topical adjuvant to reduce systemic corticosteroid use in severe OLP. The objective of the study is to develop, optimize, characterize ATV-Hyalugel and evaluate its clinical efficacy in a randomized controlled clinical trial. ATV-loaded hyalurosomes (ATV-HAs) were prepared via thin-film hydration and optimized using an I-optimal mixture design (independent variables: phospholipid, Tween 80, and hyaluronic acid; responses: entrapment efficiency (EE%), particle size (PS), and zeta potential (ZP). The optimal formulation was incorporated into a chitosan gel, which was characterized for its pH, rheological behavior, and in-vitro drug release. Four weeks randomized controlled trial (n = 90) compared: group one received standard prednisolone (40 mg/day) while group two received half-dose prednisolone (20 mg/day) in combination with ATV-Hyalugel (topically, three times daily). Pain and ulcer scores were recorded weekly. Between-group comparisons were performed using the Mann–Whitney U test (non-parametric; α = 0.05), and within-group improvement from baseline to Week 4 was assessed using the Kruskal–Wallis test. Optimized ATV-HAs demonstrated high EE% (79.1 ± 0.4%), uniform PS (221.2 ± 5.1 nm), and stable ZP (–31.6 ± 0.2 mV). ATV-Hyalugel exhibited mucosa-compatible pH (6.48 ± 0.2), pseudoplastic rheology, and a sustained release profile dominated by diffusion-driven kinetics. Clinically, group two achieved therapeutic equivalence to group one by Week 2 (p > 0.05), despite receiving 50% less corticosteroid. Both groups showed significant symptom reduction from baseline to Week four (p < 0.0001, Kruskal–Wallis). No adverse events were reported with ATV-Hyalugel. ATV-Hyalugel enables a 50% corticosteroid dose reduction while maintaining clinical efficacy. Its favorable release kinetics and safety profile support its use as an innovative adjuvant therapy for severe OLP.

## Introduction

Oral lichen planus (OLP) is a chronic inflammatory condition driven by immune dysregulation, primarily affecting the oral mucosa [1, 2]. It manifests through various clinical forms, including reticular, papular, erythematous, ulcerative, bullous, and atrophic types. While reticular and papular variants are often asymptomatic, the ulcerative and bullous forms can cause significant pain, burning sensations, and functional impairments, adversely affecting patients' quality of life [3, 4]. OLP is a chronic mucocutaneous disorder that affects approximately 0.5% to 2% of the adult population, typically between the ages of 30 and 60 years, with a higher prevalence in females and a rare occurrence in children [5]. According to multiple systematic reviews and meta-analyses [6–8], the global prevalence of OLP in the general population is estimated at 0.89% (95% CI: 0.38–2.05%) and around 1.01% in clinical populations [9–11]. More recent data from 2004 to 2024 continue to report a consistent global prevalence of approximately 1% [12, 13]. Epidemiological studies reveal notable regional variation in the general population. OLP prevalence is estimated at 0.57% in Asia, 1.68% in Europe, and 1.39% in South America. In clinical settings, higher rates have been observed: 1.43% in Africa, 3.18% in South America, and 0.11% in North America [14, 15]. Importantly, ulcerative forms of OLP tend to show poorer treatment responses [12], reinforcing the need for improved and individualized management strategies. OLP is characterized by a T-cell-mediated immune response, although its exact pathogenic mechanisms remain partially understood. Research suggests that OLP develops from a chronic dysregulated response to specific antigens presented by innate immune cells and keratinocytes [16]. This immune dysregulation leads to the overproduction of cytokines, chemokines, and adhesion molecules [17], which recruit T-cells and mast cells to the affected area, resulting in keratinocyte apoptosis and disruption of the mucosal basement membrane [18]. Managing OLP is challenging due to its multifaceted nature. Asymptomatic cases may not require intervention, but symptomatic forms, particularly erythematous and ulcerative, often necessitate treatment aimed at alleviating discomfort and promoting healing. Corticosteroids are the primary therapeutic option; however, their long-term use possesses significant limitations and potential side effects [19], including mucosal atrophy (30% of long-term users), candidiasis, metabolic complications, and high recurrence rates after withdrawal [20–22], requiring dose-sparing strategies [23]. The recurrent nature of OLP underscores the need for effective management strategies to address flare-ups and improve patient outcomes [24]. Given the variability in individual responses to steroid therapy, there is an increasing interest in exploring alternative or adjuvant treatments that minimize reliance on corticosteroids and their associated risks [19, 24]. Novel therapeutic approaches include JAK inhibitors [25, 26] and calcineurin inhibitors [27, 28], though concerns persist regarding serious adverse events such as infections and malignancy [29]. Similarly, while calcineurin inhibitors are effective, their use has been limited by long-term safety concerns, including potential carcinogenic risks [26, 30]. Recent studies have suggested that statins, primarily known for their cholesterol-lowering effects [31], may offer significant therapeutic benefits in managing OLP symptoms. These 3-hydroxy-3-methylglutaryl-coenzyme A (HMG-CoA) reductase inhibitors not only serve as a primary treatment for hyperlipidemia due to their safety, cost-effectiveness, and efficacy in reducing low-density Lipoprotein levels, but they also possess notable anti-inflammatory and immunomodulatory properties. A 2023 meta-analysis confirms statins' efficacy in oral inflammatory diseases through immunomodulation [32]. This dual action could address the underlying dysregulation seen in OLP, highlighting the need for further research into statins as a potential adjunct therapy. In addition to their lipid-lowering capabilities, statins have garnered attention for their ability to modulate immune responses, which may be beneficial in various conditions, including rheumatoid arthritis, inflammatory bowel disease, and asthma [33]. Their anti-inflammatory effects could also extend to oral health, where statins have shown promise in managing periodontal disease [34, 35]. Evidence indicates that statins can reduce gum inflammation, enhance tissue repair, and promote bone regeneration, effectively addressing concerns such as alveolar bone loss in periodontitis [36]. Atorvastatin (ATV), a widely studied statin, has emerged as a particularly relevant agent in evaluating therapeutic strategies for oral conditions like OLP [37]. Its potential to modulate inflammatory responses [38, 39] and improve oral health outcomes positions it as a key player in our investigation as an adjunct therapy for OLP management. Furthermore, the antiviral activity of statins against viruses such as hepatitis C and HIV may also be relevant, as OLP can sometimes be triggered by viral infections [40]. This connection underscores the multifaceted benefits of these medications. Several previous studies adopted topical buccal application of ATV formulations for different oral conditions, such as mucoadhesive tablets for recurrent aphthous stomatitis [41], PEGylated cubosomal in situ gel for periodontitis and mouthwash dosage forms for the prophylaxis against radiotherapy induced mucositis and denture stomatitis [42–44]. Other relevant studies reported the beneficial application of ATV topically in miscellaneous conditions other than its buccal applications, such as chronic hand eczema [45], dry eye associated with blepharitis[46], and diabetic wound healing [47].

ATV is classified as a lipophilic drug under the Biopharmaceutical Classification System (BCS), categorized as a class II drug due to its high permeability with a log P value of 5.7 [48]. While ATV effectively crosses mucous membranes, its low aqueous solubility of 0.1 mg/mL [49] results in a suboptimal oral bioavailability of less than 12%. To address these challenges, we developed atorvastatin-loaded hyalurosomes (ATV-HAs) as one of the advanced delivery systems. these advanced delivery systems are critical for topical statin efficacy, as recent reviews highlight mucoadhesive hydrogels as promising solutions [50]. The nanosystem's unique architecture offers several benefits for the oral mucosa [51]. First, it facilitates improved penetration through the mucosal barrier, ensuring a more effective therapeutic concentration at the site of action. Second, the sustained release profile of the hyalurosomes allows for prolonged drug availability, reduces the need for frequent dosing and enhances patient compliance [52], which is crucial for maximizing therapeutic effects in the oral cavity [53]. Hyalurosomes, as an advanced class of vesicular nanocarriers [54], differ significantly from conventional lipid-based systems such as liposomes and niosomes. Their distinctiveness lies in the integration of hyaluronic acid (HA) into the vesicle architecture either during hydration or as a surface-associated component, resulting in a hydrated, deformable structure with an HA-enriched corona. This configuration not only enhances mucoadhesion and prolongs mucosal residence time but also supports biological activities such as tissue hydration, anti-inflammatory action, and epithelial healing, making it particularly beneficial for oral mucosal applications [55, 56]. Moreover, HA facilitates targeted delivery by binding to CD44 receptors [57, 58], which are commonly overexpressed in inflamed oral tissues, such as those affected by OLP. Compared to conventional vesicles, hyalurosomes thus provide improved biocompatibility, mucosal targeting, and therapeutic synergy, making them particularly advantageous for the localized treatment of chronic inflammatory oral diseases.Authors prepared, characterized, and optimized ATV-HAs using an I-optimal mixture design (IOMD). The optimal formulation was further incorporated into a chitosan-based gel (ATV-hyalugel), which benefits from chitosan's mucoadhesive, biocompatible, and antimicrobial properties [59]. This synergy between ATV, HA, and chitosan is targeted at managing OLP effectively. Importantly, to validate ATV-hyalugel formulation's efficacy, the authors conducted a clinical trial aimed at demonstrating its effectiveness in alleviating the symptoms and manifestations of OLP. The ATV-hyalugel formulation could also serve as an adjuvant to systemic corticosteroids, facilitating a reduction in steroid dosage, an essential consideration in our study. Our study investigates ATV-Hyaulgel as a novel adjuvant to systemic corticosteroids, aiming to enhance local drug delivery while mitigating systemic exposure. By focusing on the innovative delivery system of hyalurosomes, this approach aligns with the principle of therapeutic escalation for refractory OLP, where combination therapies are increasingly explored to optimize efficacy and safety. By targeting mucosal lesions directly, the hydrogel complements systemic treatment addressing an unmet need for patients who failed topical monotherapy without dismissing standard care, paving the way for more effective and safer therapeutic strategies in clinical practice.

The innovation of our work lies in: the first application of HA-integrated nanovesicles (hyalurosomes) for OLP management, demonstration of 50% systemic corticosteroid reduction efficacy via a randomized clinical trial and a statistically optimized formulation overcoming statin delivery challenges.

## Materials

Atorvastatin calcium (ATV) was obtained from Epico Co. (10^th^ of Ramadan, Cairo, Egypt). L-α-phosphatidylcholine from egg yolk (PL) was purchased from Sigma-Aldrich® (St. Louis, MO). Hyaluronic acid as sodium salt (HA), 95% (Mwt; 1.5—2.2 million Da) and chitosan (Mwt; 100.000 – 300.000) were purchased from Acros Organic Co. (Geel, Belgium). Methanol, potassium dihydrogen phosphate (KH_2_PO_4_), Tween®80 (T_80_), disodium hydrogen phosphate (Na_2_HPO_4_), sodium chloride (NaCl), and glacial acetic acid were purchased from Adwic, El-Nasr pharmaceutical Co. (Abu-Zaabal, Cairo, Egypt). All other chemicals and solvents were of analytical grade and used as received.

## Methods

### Experimental design

An IOMD, using Design Expert® software (Version 13, Stat-Ease Inc. Minneapolis, MN, USA) (DX13), was employed for preparing and optimizing the ATV-HAs, followed by the analysis of variance (ANOVA) to determine the significance of each mixture component on the examined formulation responses [60]. Since prediction throughout the experimental space is the primary goal of the investigation, IOMD was selected as the mixture design of choice. After preliminary screening and investigations, IOMD was used to evaluate three mixture components (MCs): (A) PL amount (L-α-phosphatidylcholine from egg yolk) (mg), (B) Tween 80 (T_80_) amount (mg) (as an edge activator) and (C) HA amount (mg) (used as sodium salt). Table 1 displays the MCs of IOMD, their actual, real, and L-pseudo-coded values, the responses to be examined, and their constraints. ATV-HAs were prepared and assessed for the entrapment efficiency percentage (EE %) (Y_1_), particle size (PS) (Y_2_), and zeta potential (ZP) (Y_3_). The model was considered significant at *p* ≤ 0.05.

**Table 1:** I-optimal mixture design for Atorvastatin-loaded hyalurosomes preparation and optimization

**Mixture components**	**Actual values**		**Real values (proportions)**		**L-pseudo values**	
	**Lower limit**	**Upper limit**	**Lower limit**	**Upper limit**	**Lower limit**	**Upper limit**
A: lipid amount (mg)	260	300	0.813	0.938	0	1
B: T_80_ amount (mg)	10	50	0.031	0.156	0	1
C: HA concentration (%)	10	50	0.031	0.156	0	1
**Examined responses**	**Aimed constraints**					
Y_1_: EE (%)	Maximize					
Y_2_: PS (nm)	Minimize					
Y_3_: ZP (mV)	Maximize (as absolute values)					

**N.B: **T_80_: Tween^®^80, HA: Hyaluronic acid,EE %: entrapment efficiency percentage, PS: particle size, and ZP: zeta potential

### Preparation of ATV-HAs

ATV-HAs were developed using the thin film hydration method with certain modifications [61]. Briefly, a solution of specific amounts of ATV, PL and the edge activator (T_80_) was prepared using 10 mL of a mixture of chloroform and methanol as a solvent (7:3). After that, the organic solution was completely evaporated under reduced pressure until a thin film was obtained, using rotavapor (Heidolph VV, 2000, Burladingen, Germany) at a temperature of 60°C for 15 min at 150 rpm. 10 mL of an aqueous solution of different concentrations of HA was used to hydrate the previously obtained film (final ATV concentration was 2 mg/mL). Dispersions were left hydrating for 30 min at 150 rpm without vacuum, to promote the swelling of the PL and the development of ATV-HAs dispersions. The obtained ATV-HAs dispersions were then sealed and stored overnight in a refrigerator (4°C) for stabilization before further characterization. The composition of different ATV-HAs is placed in Table 2.

**Table 2:** The composition and mean results (±SD) of examined responses of different formulated Atorvastatin-loaded hyalurosomes

**Formulation**	**Phosphatidylcholine amount (mg)**	T_80_ amount **(mg)**	**HA amount (mg)**	**EE (%)**	**PS (nm)**	**ZP (mV)**	**PDI**
ATV-HA_1_	300	10	10	91.5 ± 0.7	633.6 ± 0.6	- 54.4 ± 1.5	0.60 ± 0.2
ATV-HA_2_	280	30	10	94.2 ± 5.5	680.6 ± 0.1	- 40.6 ± 2.4	0.58 ± 0.3
ATV-HA_3_	280	30	10	94.3 ± 0.9	680.6 ± 2.9	- 40.6 ± 2.4	0.86 ± 0.0
ATV-HA_4_	280	30	10	94.3 ± 0.0	600.9 ± 21.9	- 42.6 ± 0.6	0.73 ± 0.2
ATV-HA_5_	260	50	10	66.7 ± 0.8	570.8 ± 15.8	- 32.3 ± 1.8	0.58 ± 0.3
ATV-HA_6_	266.66	36.66	16.66	85.5 ± 0.1	548.0 ± 5.2	-31.2 ± 0.3	0.68 ± 0.2
ATV-HA_7_	273.33	23.33	23.33	89.6 ± 0.0	650.8 ± 0.3	- 44.9 ± 0.9	0.56 ± 0.2
ATV-HA_8_	280	10	30	82.3 ± 0.1	805.0 ± 2.8	- 56.1± 0.8	0.82 ± 0.2
ATV-HA_9_	280	10	30	82.3 ± 1.6	805.0± 2.8	- 56.1± 0.8	0.69 ± 0.2
ATV-HA_10_	260	30	30	90.9 ± 1.5	615.0 ± 14.8	- 42.0 ± 0.7	0.68 ± 0.3
ATV-HA_11_	260	30	30	90.8 ± 0.1	615.0 ± 14.8	- 42.0 ± 0.7	0.59 ± 0.3
ATV-HA_12_	266.66	16.67	36.66	88.6 ± 0.5	830.3 ± 0.0	- 46.6 ± 0.5	0.65 ± 0.3
ATV-HA_13_	260	10	50	93.2 ± 0.0	1034.0 ± 56.1	- 50.8 ± 1.1	0.70 ± 0.2

**N.B. **ATV-HAs:Atorvastatin-loaded hyalurosomes**, **T_80_: Tween^®^80, HA: Hyaluronic acid,EE %: entrapment efficiency percentage, PS: particle size, and ZP: zeta potential, PDI: Polydispersity index

### In-vitro characterization of ATV-HAs

#### Determination of entrapment efficiency percentage (EE%)

An EE% test was performed to determine how well hyalurosomes encapsulated ATV. The indirect method was applied [62, 63], involving centrifuging 1mL of each ATV-HAs formulation using a cooling ultra-centrifuge (Beckman, Fullerton, Canada) at 22,000 rpm for 1 h at 4 ˚C. After that, the supernatant was collected, properly diluted with methanol, and measured spectrophotometrically (UV/VIS spectrophotometer, Model 1800, Shimadzu, Kyoto, Japan) at a predetermined λ_max_ (247.0 nm) using a previously constructed calibration curve, for which limit of detection (LOD) and limit for quantification (LOQ) were calculated. Each measurement was done three times, and the results were computed accordingly:

1 $$EE\;\%\;=\frac{Theroetical\;amount\;of\;ATV\;-\;unentrapped\;amount\;of\;ATV}{Theroetical\;amount\;of\;ATV\;}\times100$$

### Determination of particle size (PS), polydispersity index (PDI), and zeta potential (ZP)

A zeta-sizer nano ZS (Model ZEN3600, Malvern Instrument LTD., Worcestershire, UK) was used to measure the PS, PDI, and ZP of the prepared ATV-HAs [64]. Each sample was measured three times at 25°C after being suitably diluted with deionized water. PS was measured by the dynamic light scattering technique [65]. ZP measurements were conducted by observing the electrophoretic mobility of ATV-HAs in the electric field using the same apparatus [66]. Mean values of triplicate results were also recorded (± SD).

### Analysis and optimization of ATV-HAs

After the IOMD results analysis, the desirability function was applied, by DX13, to pick an optimum ATV-HAs formulation. This optimum ATV-HAs formulation was picked according to selection criteria, including minimizing PS and maximizing both the EE% and ZP (as absolute values). All the examined responses were assigned the same importance (3 +). The desirability function implemented can be described by the following equation [61]:

2 $$D={(d_1.d_2\dots d_m)}^\frac1m$$

D: overall desirability, d_m_: the desirability of the m^th^ response, ranging from 0 to 1.

The optimum ATV-HAs formulation was sonicated for further size reduction in an ice bath for 2 min (five seconds on and five seconds off) using a probe sonicator (Model UP-500, 220V, ChromTech, Apple Valley, Minnesota, USA), and its EE%, PS, ZP, and release profile were evaluated.

### In-vitro characterization of optimum ATV-HAs formulation

#### Transmission Electron Microscopy (TEM)

The optimum ATV-HAs formulation morphology was assessed using TEM (Model JEM-1230, Jeol, Tokyo, Japan). One drop of the unstained dispersion was applied to a carbon-coated copper grid and left to dry for 10 min at room temperature before being examined under a TEM operated at 80 kV [63].

#### Preparation of ATV-hyalugel

Chitosan (100 mg) was sprinkled on 10 mL of the optimum ATV-HAs formulation (containing 20 mg of ATV); then the pH was rendered acidic by adding a few drops of 1% v/v acetic acid solution. The mixture was blended via a magnetic stirrer (WiseStir, Daihan Scientific Co., Ltd., Korea) at 25°C. The ATV-Hyalugel was further evaluated.

### In-vitro evaluation of ATV-hyalugel

#### pH measurement

One gram of ATV-hyalugel was diluted with 9 mL of distilled water. Then its pH was measured by immersing the electrode of a Jenway pH meter (model-3505, Bibby Scientific Ltd., Stone ST15 0SA, UK) into the diluted hyalugel for a minute to acclimate, and the average pH value was calculated [62].

#### In-vitro release studies

The release of ATV from ATV suspension and the optimum ATV-HAs, in comparison to its corresponding hydrogel (ATV-hyalugel), was studied using the dialysis bag diffusion technique [67]. To ensure sink conditions, the release media was 50 mL of hydro-alcoholic solvent, composed of (5% methanol with 95% PBS (pH 7.4)). The experiment was conducted in a thermostatically controlled shaking water bath (Maxturdy-30, Witeg Labortecnik, Gmbh, Germany) maintained at 35 ± 0.5˚C. One mL of ATV-HAs (containing 2 mg ATV) was placed in a tightly closed cellulose dialysis membrane (diameter 25 mm, MW 12,000–14,000 Laboratories Inc., CA, USA) and immersed in a bottle containing 50 mL of the release media. The thermostatically controlled Shaker was adjusted at 100 strokes per minute, and at several time intervals over 24 h, samples of 3 mL from the release medium were collected and assayed spectrophotometrically at the predetermined λ_max_ (247.0 nm). Each withdrawn sample was replaced by an equal volume of the fresh release medium, and each experiment was done in triplicate. The same conditions were applied for ATV-hyalugel. The cumulative amount released at different intervals was calculated according to the following formula [68]:

3 $${Q}_{n}=\frac{\left({C}_{n}\times {V}_{r}+{\sum }_{i=1}^{n-1}{C}_{i}\times {V}_{s}\right)}{Initial\ drug\ amount}\times 100$$

where Q_n_ represents the percentage of cumulative ATV released, C_n_ is the concentration of the receptor release medium at n^th^ sample, V_r_ denotes the total volume of the receptor release medium, V_s_ denotes the volume of each sample withdrawn for determination and ${\sum }_{i=1}^{n-1}{C}_{i}$ is the summation of the previous concentrations.

Kinetic analysis of ATV release from ATV suspension, the optimum ATV-HAs, and ATV-hyalugel was employed using zero-order and first-order models. The model showing the maximum linearity, i.e. the highest R^2^, will be the model of choice. Each release mechanism was also analyzed according to Higuchi diffusion and the Korsmeyer-Peppas models [61]. The mathematical modeling and equations were processed using Microsoft Excel (Microsoft Office 365, One Microsoft Way, Redmond, Washington, USA), and R^2^ was calculated for each. The Zero-order model was described by a plot of (Qn) versus (time), and the first-order model was drawn as a plot of (log % remaining (log (100-Qn)) versus (time) [69, 70]. As for the Higuchi diffusion pattern, (Qn) was drawn versus (√ time). Regarding Korsmeyer-Peppas, (log Qn) was drawn versus (log time), and the release exponent (n) was calculated to identify the mechanism by which ATV was released from ATV suspension, the optimum ATV-HAs formulation, and ATV-hyalugel [71].

The following equations describe the applied mathematical models [70]:

Zero order:

4 $${Q}_{n=} {Q}_{0}+{k}_{0}t$$

First-order kinetics:

5 $$log \left(100-{Q}_{n}\right)=log{Q}_{0 }-\frac{kt}{2.303}$$

Higuchi diffusion:

6 $${Q}_{n}={k}_{H} \surd t$$

Korsmeyer-Peppas:

7 $$log{Q}_{n}=logk+n\mathrm{log}t$$

#### Rheological assessment of ATV-hyalugel

The viscosity of the ATV-hyalugel was measured at 25 °C ± 0.1 °C, using a Brookfield viscometer (Model HBDV-1 + CP, Spindle CPE-41, Middleboro, MA, USA). ATV-hyalugel was added to the plate in an amount of 1 g. The shaft was rotated in a range from 0.5 to 100 rpm, and the viscosity results were recorded only for torque values ranging from 10 to 100% [72]. The relationship between viscosity, shear stress, and shear rates was plotted. The rheological behavior was investigated using the power law model:

8 $$\tau =k{\gamma }^{n}$$

τ: the shear stress.

k: the consistency index.

γ: the shear rate.

n: the flow index.

The system of the flow pattern is evaluated by the n value. Newtonian systems are recognized when n = 1, while n values < 1 indicate shear thinning systems. When n > 1, the system is dilatant. To examine the flow pattern, the rheological data were fitted to the non-Newtonian Bingham’s, Carreau’s and Casson’s models [62]. The flow pattern of the examined ATV-hyalugel was represented by the model exhibiting the greatest R^2^ value.

#### Dynamic light scattering (DLS) and zeta potential measurements of ATV-hyalugel

To directly assess vesicle integrity post incorporation, ATV-Hyalugel and blank chitosan hydrogel (1% w/v, without vesicles) were diluted in distilled water (0.1 mL in 10 mL deionized water). DLS and ZP measurements were performed using a Malvern Zetasizer Nano ZS (Malvern Panalytical, UK) at 25 °C. Samples were vortexed for 60 s prior to analysis to ensure homogeneity. For DLS, 1 mL aliquots were transferred to disposable sizing cuvettes. ZP measurements were conducted by observing the electrophoretic mobility of ATV-Hyalugel in the electric field using the same apparatus [66]. Mean values of triplicate results were also recorded (± SD).

### Clinical study design

#### Patient’s grouping

This study was a randomized control clinical trial, approved by the Institutional Review Board (MUST-IRB) of research ethics (Approval number:2024/0060), MUST-IRB is registered at the Office for Human Health Protection, US Department of Health and Human Services and operates under Federal Wide Assurance No. FWA00025577. The study protocol was also approved by the Research Ethics Committee (REC) for experimental and clinical studies at the Faculty of Pharmacy, Cairo University, Approval number: PI (3854).

A power analysis calculated sample size was designed to have adequate power to apply a two-sided statistical test of the null hypothesis that there is no difference between the tested groups regarding pain severity. By adopting an alpha (α) level of 0.05 (5%), a beta (β) level of 0.2 (i.e. power = 80%) and an effect size (d) of 0.62, calculated based on the results of a previous study [73]. The predicted total sample size (n) was found to be 90 cases (i.e. 45 cases per group). Sample size calculation was performed using G*Power version 3.1.9.7. Participants for the study were recruited from the outpatient clinic of the oral diagnosis clinic of the faculty of oral and dental surgery at Misr University for Science and Technology. based on specific inclusion and exclusion criteria.

Inclusion criteria required participants to have a prior clinical and histological diagnosis of severe OLP, currently in an exacerbation phase. Eligible individuals had to be willing to participate in the study and be between 30 and 50 years old, regardless of gender. Conversely, individuals were excluded from participation if they were immunocompromised, pregnant, or nursing. Additional exclusion criteria included current smokers or alcohol consumers, those with any dermatological diseases, endocrine dysfunctions, history of a malignancy, individuals undergoing hormone replacement therapy, those taking steroid medications, or those who had consumed non-steroidal anti-inflammatory drugs (NSAIDs) within the four weeks preceding the study. This structured approach ensured a focused participant selection aligned with the study's objectives. Discontinuity criteria were also included in this clinical trial upon patient requests or for those who missed any pre-scheduled visits. This randomized controlled clinical trial was of two arms in parallel groups with a 1:1 allocation ratio. The participants' allocation into the two groups was done using a prepared sealed envelope. Each patient was asked to select an envelope to assign to the control/study group, and this allocation was done by one of the operators. According to the nature of the study, the operator who recorded the clinical examination of OLP and the statistician were blinded. Ninety participants were randomly and equally divided into two groups: a control group (Group 1) and a study group (Group 2). The control group received 40 mg of systemic corticosteroids (prednisolone, standard OLP steroids therapy), while the study group received 20 mg of prednisolone in conjunction with topical ATV-Hyalugel. All participants in both groups administered prednisolone at varying doses, with careful monitoring for potential side effects until clinical remission was achieved. Following the attainment of remission, a gradual withdrawal of corticosteroids was implemented. The dose of prednisolone was tapered progressively in both groups until the size of the lesions was reduced by 50%. Subsequently, the dosage was incrementally reduced by 10 mg each Week, culminating in a final dose of 5 mg daily during the last week of treatment. This structured approach ensured effective management of the participants while minimizing corticosteroid-related complications [74].

#### Treatment protocol

The treatment protocol in this study aimed to evaluate the efficacy of 0.2% ATV-Hyaulgel as an adjunctive therapy to 20 mg systemic corticosteroids in managing oral mucosal lesions. The protocol included comprehensive instructions for participants on maintaining optimal oral hygiene, controlling dental plaque, and managing infections, including candidiasis. Both groups received a 2% miconazole oral gel four times daily for one week each month to prevent Candida infections [75]. The study group was treated with systemic corticosteroids and ATV-Hyaulgel three times daily on affected mucosal lesions, with specific instructions for patients to dry the application area, apply a thin layer of gel, and avoid eating for one-hour post-application. Weekly follow-up visits will be conducted to assess remission status, with total ulcer scores recorded at baseline and during each visit to compare the effects of systemic corticosteroids alone versus the combination therapy. This protocol is designed to enhance patient outcomes through integrated treatment strategies while ensuring thorough monitoring of both efficacy and safety.

### Clinical parameters assessment

A scheduled follow-up was done with the patients for 1 to 4 weeks. In the context of this clinical investigation, a comprehensive assessment of oral disease severity and patient comfort was conducted using validated scoring systems and patient-reported outcomes. The Visual Analogue Scale (VAS) was utilized to measure the patient's subjective pain experience, with scores ranging from 0 (no pain) to 10 (unbearable pain). Patient satisfaction regarding overall treatment was evaluated through categorical responses of “very satisfied,” “somewhat satisfied,” or “not satisfied.” Additionally, the Oral Disease Severity Score (ODSS) serves as a critical tool for evaluating the severity of oral manifestations associated with conditions such as pemphigus vulgaris (PV), mucous membrane pemphigoid (MMP), and OLP [76, 77]. This detailed scoring system encompasses 17 distinct oral mucosal sites, allowing for a nuanced understanding of disease activity. The ODSS assigns activity scores based on the presence and extent of lesions, and scoring took place as follows: Buccal Mucosa: A score of 1 if less than 50% of the area is affected. A score of 2 means more than 50% of the area is affected. Dorsum of the Tongue, Floor of Mouth, Hard and Soft Palate, Oropharynx: A score of 1 for unilateral lesions and a score of 2 for bilateral lesions. Lesion severity is further classified into activity scores: 1 for mild erythema, 2 for marked erythema without erosion, and 3 for erosion or ulceration [78]. Additionally, the Visual Analogue Scale (VAS) was utilized to measure the patient's subjective pain experience, with scores ranging from 0 (no pain) to 10 (unbearable pain). Patient satisfaction regarding overall treatment was evaluated through categorical responses of “very satisfied,” “somewhat satisfied,” or “not satisfied.” Adverse effects associated with treatment were meticulously documented. Throughout the four-week follow-up period, outcome measures were assessed weekly. Statistical analysis focused on two primary objectives: between-group comparisons at each timepoint used Mann–Whitney U tests (non-parametric; α = 0.05), and within-group improvement from baseline to Week 4 used Kruskal–Wallis tests. This approach optimally addressed our clinical questions about treatment equivalence during critical healing phases (Weeks 2–4) and the magnitude of symptom reduction after the full treatment course.

### Analysis of data

Data was collected and analyzed in collaboration with a statistician. Following the analysis, participants were informed about the efficacy of both treatment strategies applied in this study through a scheduled meeting with all study participants.

## Results and discussion

### Development of ATV-HAs

Hyalurosomes are innovative nanovesicles that integrate the inherent properties of phospholipids (PL) and hyaluronic acid (HA). This combination imparts the durability characteristic of core gel nanocarriers while retaining the advantages of deformable liposomes [79]. The thin film hydration technique was effectively employed to prepare ATV-HAs, achieving satisfactory drug entrapment, appropriate size, and high physical stability. A preliminary investigation of various lipid types and edge activators led to the selection of PL from egg yolk and T_80_ for further studies. Subsequently, an IOMD was utilized to analyze the impact of MCs, such as the amounts of PL, T80, and HA, on the characterization of the resulting ATV-HAs. The results indicated that the EE% of ATV was notably high. Additionally, the nanovesicles exhibited a range of sizes and polydispersity indices, demonstrating their physical characteristics. The ZP of the ATV-HAs reflected a range of values, suggesting variations in their surface charge properties. To further investigate the influence of the components examined on the ATV-HAs, an ANOVA test was performed. The statistical analysis provided insights into the interactions between the formulation variables and the resultant properties of the nanovesicles. The analysis of each MC will be discussed separately to provide a comprehensive understanding of its individual effect on the formulation.

### Outputs of IOMD

IOMD is an effective statistical design for identifying how formulation components could influence the prepared ATV-HAs. IOMD evaluation is shown in Table 3. Generally, adjusted R^2^ reflects the fitting of the model to the currently investigated data, predicted R^2^ provides a clear image of a high-quality model and how its accuracy is suitable for predicting forthcoming data, and the adequate precision indicates the signal-to-noise ratio, it ensures that the model utilized for each examined response can navigate the design space [80]. The IOMD evaluation data in Table 3 showed that all adequate precision values were more than 4. Furthermore, it showed the agreement between the adjusted R^2^ and the predicted R^2^ values as the difference between both values for the same response is not more than 0.2.

**Table 3:** Model evaluation data of the I-optimal mixture design for Atorvastatin-loaded hyalurosomes preparation.

**Output**	**Responses**		
	Y_1_:EE (%)	Y_2_:PS (nm)	Y_3_: ZP (mV)
Model type	Special cubic	Reduced quadratic	Reduced quadratic
RMSE	0.5091	37.18	2.92
CV%	0.5784	5.31	6.64
R^2^	0.9978	0.9431	0.8878
Adjusted R^2^	0.9956	0.9242	0.8504
Predicted R^2^	0.8628	0.8936	0.7725
Adequate precision	73.5	20.7	14.6
*P* value	< 0.0001	< 0.0001	0.0001

**N.B:**EE: entrapment efficiency percentage, PS: particle size, ZP: zeta potential, RSME: root mean square error. When p-values are < 0.05, this indicates significant models

### Characterization of ATV-HAs and the influence of MCs on the examined responses

**Fig. 1:**
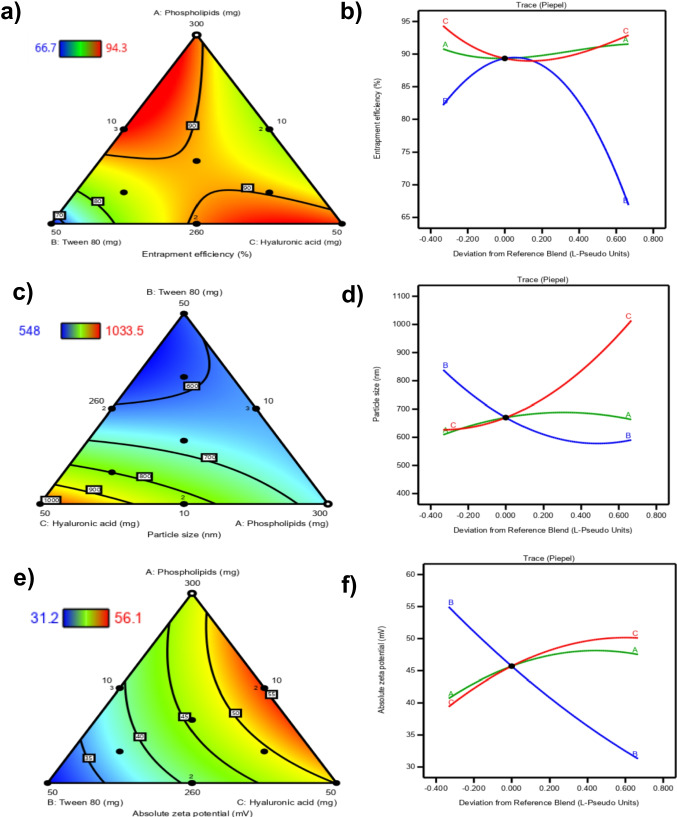
Contour plots, (**a**, **c**, and **e**) and Piepel’s plots (**b**, **d**, and **f**) demonstrating the influence of varying the proportions of the mixture components (A, B and C) over the I-optimal design space, on the percentage entrapment efficiency (**a** and **b**), the particle size (**c** and **d**), and the absolute zeta potential (**e** and **f**) of Atorvastatin-hyalurosomes

For a mixture design, the effect of MCs on the responses examined can be represented by contour and piepel plots as shown in Figs. (1a, 1c, and 1e) and Figs. (1b, 1d, and 1f), respectively. Piepel plots (trace plots) are commonly used to describe the MCs' effects. The reference blend point (REF) is the chief point in the piepel plot where the three MCs are equal in values (1/3, 1/3,1/3). A piepel plot for a response is a plot of L-pseudo values’ deviations from REF for each MC on the X-axis versus the examined response on the Y-axis, where the L-pseudo values for each MC, ranging from 0 to 1, are the converted original values of the MC to a fraction of the blend [60].

#### EE% model analysis

EE% was quantified using the UV spectrophotometry method. The calibration curve, added to the supplementary data, showed a good linearity (R^2^ = 0.9996) with LOD = 0.769 µg/mL and LOQ = 2.33 µg/mL. As listed in Table 2, values of EE% of ATV-HAs ranged from 82.30 ± 1.8% to 94.3 ± 0.9%. ANOVA, carried out by DX13, showed that the model was significant (*p* < *0.0001*), and the regression equation for EE% was as follows:

9 $$EE\left(\%\right)= + 91.53A+66.89 B+92.89 C+60.52 AB-40.20 AC+43.60 BC-41.55 ABC$$

As shown in Fig. 1b, component A (PL) positively affected the EE% of nanovesicles, i.e. an increment in ATV EE% was observed with the increasing amounts of PL, as shown in Table 2. The increase in EE% of ATV with higher phospholipid amounts highlights a complex interplay of factors that extend beyond basic geometric considerations. Elevated PL levels enhance drug-lipid interactions, which are crucial for the effective solubilization of such hydrophobic ATV (log P = 5.7) [48]. This increased hydrophobicity can lead to improved retention of the drug within the hyalurosomes, as the affinity between ATV and the lipid matrix is strengthened. Additionally, the stability of the nanovesicles is significantly impacted by PL concentration; while moderate increases can enhance bilayer integrity and reduce leakage, excessive PL may disrupt vesicle stability and lead to aggregation, adversely affecting EE%. Therefore, the relationship between PL content, encapsulation efficiency, and vesicle stability is intricate, necessitating a careful balance that optimizes drug retention while ensuring the structural integrity of the nanovesicles. Similar results were obtained by Makled et al. [79] as an increased lipid: Melatonin ratio resulted in higher EE%. Naguib et al. [81] also reported that an increment in EE% accompanied the elevation of the PL amount in Ganciclovir-loaded vesicles. Similarly, the introduction of more PL to HA-modified Agomelatine bilosomes enhanced their EE% values [82].

Figure 1b also illustrates that component B (T_80_) had a positive parabolic effect on EE%; as the increment in T_80_ amount resulted in EE% values elevation till the REF point, after which a decline in EE% was observed. T_80_ as a surfactant is commonly used in drug delivery systems to stabilize nanovesicles, reduce the chance of aggregation and improve the EE% [83]. Besides, it might increase ATV solubilization, helping to encapsulate higher amounts. After REF, a further increase in T_80_ amounts led to a lower EE% because of the probable increased hyalurosomal membrane fluidity, leading to ATV diffusion from ATV-HAs. These findings were like those stated by Ishak et al. [84] as high concentrations of T_80_ significantly lessened the EE of Rutin-loaded nanoparticles. They also coincide with the results observed by AbdelHakeem et al. [61] and Janga et al. [85] as a further increase in the surfactant amounts resulted in Itraconazole and Natamycin leakage from nanovesicles, respectively.

As for component C, HA improved the ATV EE%. As displayed in Fig. 1b, higher amounts of HA led to a significantly higher ATV EE%. This could be explained by the existence of HA long chains with repeated units of sugar that can protrude from the vesicles and enhance steric hindrance against drug egress from hyalurosomes, hence increasing ATV EE% [49]. In their investigations, Kawar and Abdelkader [86] found that the addition of HA to liposomes led to similar results. They discovered that HA produced more stable vesicles with a low possibility of aggregating and a decreased tendency to leak the encapsulated Ketoprofen. Similar observations were found by Eladawy et al. [63] as the HA increment led to higher Diacerein entrapped in the formed Hyalurosomes.

#### PS model analysis

In pharmaceutical formulation studies involving lipid-based formulations, the concentrations of various components play a critical role in determining the PS of the prepared systems. PS is an important parameter affecting the stability, release behavior and effectiveness of nanovesicles, especially for topical preparations [87]. PS of ATV-HAs, listed in Table 2, ranged from 548.0 ± 5.2 nm to 1034.0 ± 65.1. ANOVA demonstrated that the model was significant for PS (*p* < 0.0001). The regression equation for PS was as follows.

10 $$PS\;\left(nm\right)\;=\;+\;663.20\;A\;+\;590.36\;B\;+\;1013.90\;C\;-\;772.65\;BC$$

By inspecting component A (PL), Fig. 1d showed that increasing PL amounts led to ATV-HAs’ PS expansion. This can be attributed to the formation of more aggregates with higher PS values. Similar findings were earlier mentioned by Chen et al. [88], AbdelHakeem et al. [61], and Saha et al.[89] as the increment of PL amount in different nanocarriers led to an enlarged PS. Shaker et al. [90] also reported that increasing Phosal® 53 MCT concentrations produced liposomes with larger PS. They explained their findings in terms of viscosity elevation of the formed dispersions.

Regarding component B (T_80_), Fig. 1d reveals that increasing the amount of T_80_ tends to reduce PS, leading to smaller and more stable systems due to the reduction of surface energy and vesicular growth avoidance [91]. This could be related to the fact that T_80_ facilitates the formation of smaller droplets by lowering the interfacial tension between the two different phases, resulting in finer vesicles. Those results are in harmony with what was reported by Sukmawati et al. [91] as high T_80_ concentrations resulted in a drop in the PS of the nanostructured lipid carriers. Liang et al. [92] also suggested that hydrophobic interaction between T_80_ and hydrophobic drugs causes the nanoparticle core to tighten, resulting in a decrease in particle size [93].

As for component C (HA), Fig. 1d shows that HA triggered the formation of vesicles with a larger PS. This is probably due to HA's ability to bind with the choline groups of phosphatidylcholine moieties of the PL, and this may encourage the formation of larger ATV-HAs [94]. It was also reported that HA can intercalate into the PL bilayer, leading to PS expansion besides being adsorbed on the vesicular surface [95]. This result agreed with previous findings reported by Manca et al. [96]. They investigated HA concentrations ranging from 0.1% to a maximum of 0.5% to avoid larger Curcumin-loaded hyalurosomes. Castangia et al. [95] and Elsheikh et al. [97] also found that HA incorporation in liquorice extract-loaded hyalurosomes and caffeine-loaded hyalurosomes led to PS enlargement, respectively.

PDI results, as displayed in Table 2, ranged from 0.56 ± 0.2 to 0.86 ± 0.0. Dispersions with PDI values from 0.08 to 0.7 are mid-range polydisperse systems, and dispersions with PDI values ≥ 0.8 are considered highly polydisperse[98].

#### ZP model analysis

ANOVA showed that the model for ZP was significant (*p* = 0.0001). The regression equation for ZP (mV) was as follows:

11 $$ZP\;\left(mV\right)=\;+\;47.52\;A\;+\;31.29\;B\;+\;50.10\;C\;+\;24.61\;AC$$

The stability of nanodispersions is largely dependent on ZP. Formulations with ZP values > 30 mV are regarded as stable [60]. This could be explained by the repulsive forces that stop particles from aggregating and coalescing. The stability of ATV-HAs was demonstrated by the negative surface charges present in all formulations, ranging from – 31.2 ± 0.3 mV to – 56.1 ± 0.8 mV, as illustrated in Table 2. This complements the earlier findings published by Manca et al. [96] and Makled et al. [79]. Concerning ZP, all ATV-HAs were characterized by their negative ZP values. This result may be attributed to the phosphatidylcholine content of PL. It is a zwitterion carrying both phosphate and choline moieties. At pH = 7.4, negatively charged phosphate groups predominate and increase negative charges on the vesicular system surface [94]. This can explain the positive effect of component A (PL) on ZP, as displayed in Fig. 1f. Increasing PL led to an increase in the magnitude of ZP due to more phosphate groups being involved in charge generation.

On the other hand, Fig. 1f clearly showed that by increasing component B (T_80_), a subsequent decrease in ZP occurred. This may be because T_80_ is a non-ionic surfactant, so its adsorption on vesicular surfaces led to an overall decrease in the charge intensity and, hence, ZP [99]. Those findings agreed with Kheradmandnia et al. [100]. They found that increasing T_80_ concentrations led to a decline in Ketoprofen-loaded nanoparticles' ZP values. Witayaudom et al. [99] also reported similar results, as the increase in T_80_ concentration from 0.025 to 1.0% reduced the ZP of nanostructured lipid carriers.

As for component C (HA), an increase of ZP, illustrated in Fig. 1f, was observed with increasing HA amounts owing to its polyanionic nature. These results align with those observed by Eladawy et al. [63] and Elsheikh et al. [97] who reported higher ZP upon HA increment in Diacerein-loaded hyalurosomes and caffeine-loaded hyalurosomes, respectively. Notably, ZP elevation can be considered an indicator of HA adsorption on the vesicular surface [94].

### Selection of an optimum ATV-HAs formulation

**Fig. 2:**
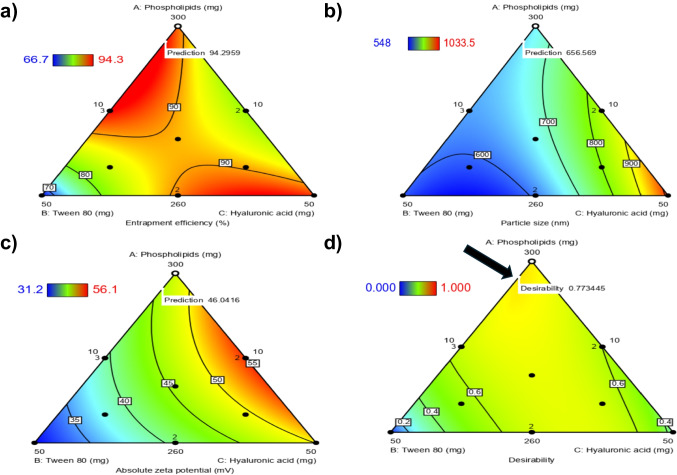
Contour plots demonstrating changes that occurred over the I-optimal mixture design space in **a**) Entrapment Efficiency (%), **b**) Particle Size (nm), and **c**) Absolute zeta potential (mV) responses, in addition to **d**) the optimum Atorvastatin-hyalurosomes formulation selected, denoted by the black arrow, and its desirability. N.B. The predicted values of each response for the optimum formulation are also shown

One optimum ATV-HAs formulation (OF) was designated based on the previously mentioned selection criteria, which included maximizing EE and ZP while minimizing PS. Numerous formulations (solutions) were offered by DX13 based on the importance provided to each examined response. A proposed formulation with the highest desirability (0.77), shown in Fig. 2, was selected. It consisted of PL (296.35 mg), T_80_ (13.64 mg), and HA (10 mg). The OF was predicted to have a PS of 656.57 nm, a ZP of – 46.04 mV, and an EE of 94.3%. Following optimization, the selected OF was subjected to sonication to reduce particle size before gel incorporation and accordingly, a re-evaluation for EE%, PS, and ZP was pivotal. After sonication, an adequate PS (221.2 ± 5.1 nm) was obtained. EE% and ZP decreased to 79.1 ± 0.4% and -31.6 ± 0.2 mV, respectively. These results were satisfactory enough to guarantee vesicular penetration through the oral mucosa and successful drug delivery, as well as the formulations’ stability (ZP was still > ± 30). The resulting sonicated OF was used for ATV-hyalugel preparation and subsequent evaluations.

#### TEM

**Fig. 3:**
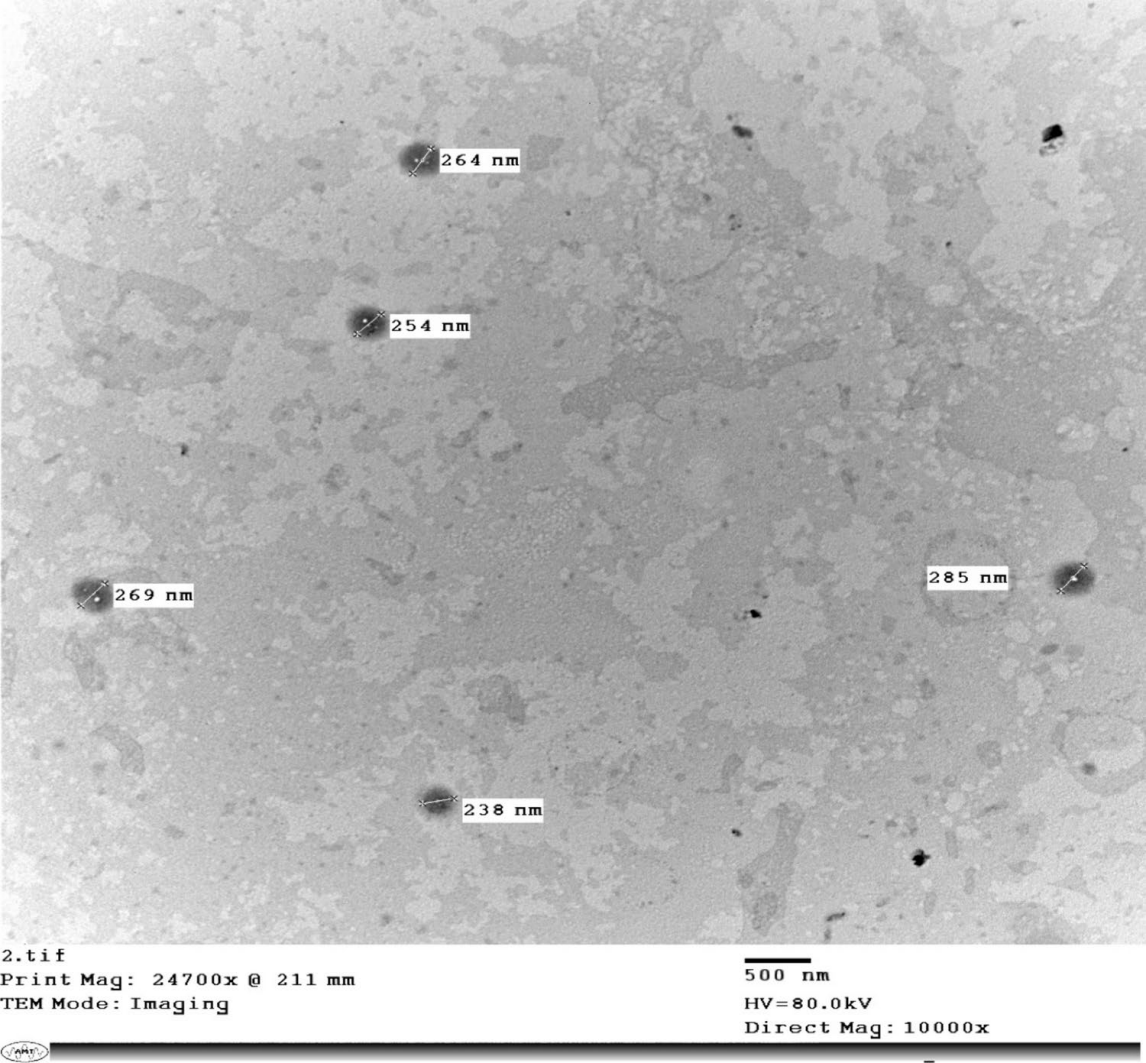
TEM photomicrograph of the optimum ATV-loaded hyalurosomes

TEM was used to perform a morphological analysis of the OF. As shown in Fig. 3, the TEM micrograph demonstrated that the optimum ATV-HAs formulation appeared as small, well-defined spherical vesicles devoid of aggregations or fusion clusters.

### The preparation of ATV-Hyalugel

The prepared ATV-Hyalugel showed promising characteristics, with the gel exhibiting a visually clear and normal appearance following the incorporation of chitosan (1% w/v) into the OF and pH adjustment. These observations suggested a favorable environment for further characterization.

### Evaluation of the prepared ATV-Hyalugel

#### pH

For a hydrogel formulation to be suitable for oral application, pH assessment is essential to avoid irritation of the oral mucosa. Physiological saliva pH normally ranges from 6.2 to 7.6, with an average value of 6.7 [101]. The produced ATV-hyalugel had an average pH of 6.475 ± 0.2, indicating that it was appropriate for oral mucosa application without irritation.

#### In-vitro drug release study

**Fig. 4:**
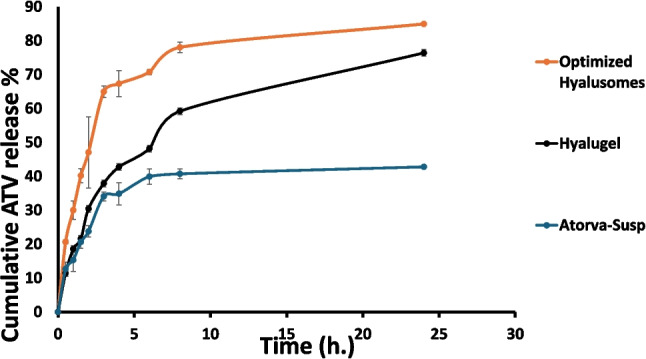
The in-vitro release profile of ATV from optimum ATV-loaded hyalurosomes, its corresponding hydrogel, and ATV-suspension

The release profile of ATV from the OF, ATV-hyalugel and ATV suspension reveals significant insights into their pharmacokinetic behaviors. As illustrated in Fig. 4, both OF and ATV-hyalugel demonstrated a gradual and sustained release over 24 h, characterized by a biphasic pattern. This pattern is indicative of an initial rapid burst release, which can be attributed to the hydrophobic nature of ATV, the drug’s affinity for the hydrophobic regions of hyalurosomes likely facilitates this initial release phase, allowing for quick therapeutic action. Furthermore, the presence of HA plays a crucial role in modulating the release profile. HA not only acts as a carrier but also serves to shield ATV particles from degradation [102]. This protective mechanism effectively prolongs drug release, as evidenced by the cumulative percentage of ATV released after 24 h, 84.9 ± 1.5% from the OF compared to 76.4 ± 1.7% from ATV-hyalugel, while that of ATV-suspension was 42.79 ± 1.43%. The substantial difference in percent cumulative of ATV release from both formulations might be due to the hydrogel matrix, which contributes as an additional layer of complexity to the release dynamics, similar results were observed by Elgendy et al. [103] The lower cumulative amount released from suspension could be attributed to its lack of structural components that facilitate controlled release [104]. Consequently, the ATV suspension may lead to suboptimal therapeutic outcomes, highlighting the importance of ATV-hyalugel to be applied in clinical study.

Kinetic modeling further elucidated the kinetic behavior and the mechanisms governing ATV release. All formulations showed a first-order release pattern, possessing higher R^2^ values than those of zero order as shown in Table 4. All formulations adhered to the Higuchi diffusion mechanism (highest R^2^ value), as shown in Table 4, demonstrating the relevance of diffusion processes in the drug release. The Korsmeyer-Peppas model analysis revealed high R^2^ values for both formulations, indicating a strong correlation with this model. The exponent (n) values, less than or equal to 0.5, suggest a Fickian diffusion. While 0.5 < n < 1.0 reflects a non-Fickian transport mechanism [105]. In this study, OF and ATV-hyalugel had exponent (n) 0.4939 and 0.5874, respectively, as illustrated in Table 4. This implies that the release of ATV from OF followed Fickian diffusion, while its release from ATV-hyalugel is governed not solely by diffusion but also by the concurrent processes of polymer swelling and erosion within the hydrogel matrix [106, 107]. For ATV suspension, the release of ATV from its suspension had also been shown to adhere Fickian diffusion mechanism (n = 0.4698). This is common in the release of insoluble drugs from their suspensions, where the diffusion mechanism predominates [108].

**Table 4:** Kinetics analysis of the release behavior of Atorvastatin from the optimum Atorvastatin-loaded hyalurosomes, its corresponding hydrogel and Atorvastatin suspension

**Formulation**	**Zero order**	**First order**	**Higuchi diffusion**	**Korsmeyer-Peppas**	**(n) exponent for Korsmeyer-Peppas **
	R^2^	R^2^	R^2^	R^2^	
Optimum ATV-HAs	0.8152	0.9063	0.9198	0.9536	0.4939
ATV-hyalugel	0.9336	0.971	0.9835	0.9828	**0.5874**
ATV-suspension	0.84	0.8454	0.9307	0.9583	0.4698

It is important to mention that Higuchi diffusion is considered a special case of the Korsmeyer-Peppas model when n = 0.5. So that the results of the two mathematical models are strongly correlated. The non-Fickian release pattern of ATV from ATV-hyalugel indicates that the therapeutic action of ATV can be finely tuned by manipulating the formulation components, particularly the amount of HA and the characteristics of the hydrogel. This control over the release profile is crucial for developing effective drug delivery systems that ensure sustained therapeutic levels over extended periods, potentially improving patient compliance and therapeutic outcomes.

#### Rheological study

**Fig. 5:**
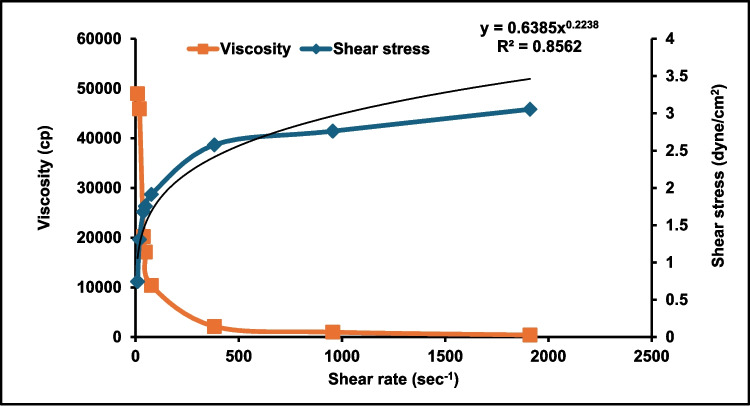
Rheological properties of the optimum Atorvastatin-loaded hyalurosomes hydrogel

Figure 5 shows that ATV-hyalugel has non-Newtonian shear thinning behavior. The power law model was used to derive the flow index (n) value, which was determined to be less than 1 (n = 0.2238). Among the investigated non-Newtonian models, Carreau’s model exhibited the highest R^2^ value (0.9947), compared to Bingham’s (R^2^ = 0.652) and Casson’s (R^2^ = 0.7305). This implies a pseudo-plastic flow, which is appropriate for topical application [72]. It is critical to highlight that pseudoplastic flow is a common feature of semisolids carrying nanosystems. This is crucial for topically applied semisolids to have their viscosities decrease with gentle application to the site of action [109].

#### DLS and zeta potential measurements of ATV-hyalugel

Direct DLS characterization of the hydrogel matrix confirmed vesicle integrity post incorporation. The loaded hydrogel exhibited a distinct nanoparticle population at 294.2 ± 12.5 nm, absent in blank hydrogel (607.1 ± 41.6 nm) with zeta potential reversal (-37.2 to + 58.1 mV) evidencing chitosan coating without vesicle disruption. This size increase reflects surface polymer adsorption, consistent with cryo-TEM observations of intact vesicles within the hydrogel network. The loaded gel size remains optimal for mucosal delivery, enabling deep epithelial penetration while avoiding systemic absorption.

### Clinical study

#### Results of the clinical study

As presented in Table 5, the statistical analysis of the baseline data for the two groups revealed no significant differences in age, pain scores, or ulcer scores, confirming the successful randomization of ninety patients. The treatment duration was four weeks, and the primary outcomes pain scores and ulcer scores were measured at the end of each week.

**Table 5:** Statistical comparison of baseline data of the control group and study group using Mann-Whitney test

**Parameters**	**Control group**	**Study group**	*P*-value
Age	50 (44.50, 55.00)	48 (44.00, 55.00)	0.5709
Pain score	7 (5.000, 8.000)	6 (5.000, 8.000)	0.7084
Ulcer score	55 (51.00, 62.00)	55 (51.50, 59.00)	0.8037

At the end of Week 1, both pain and ulcer scores significantly decreased in the control group compared to the study group, with scores of 3 (2, 4) for pain and 40 (37.5, 50) for ulcer scores. The significant difference in the control group regarding pain and ulcer scores can be correlated with the higher doses of corticosteroids administered in this group rather than the study group.

**Fig. 6:**
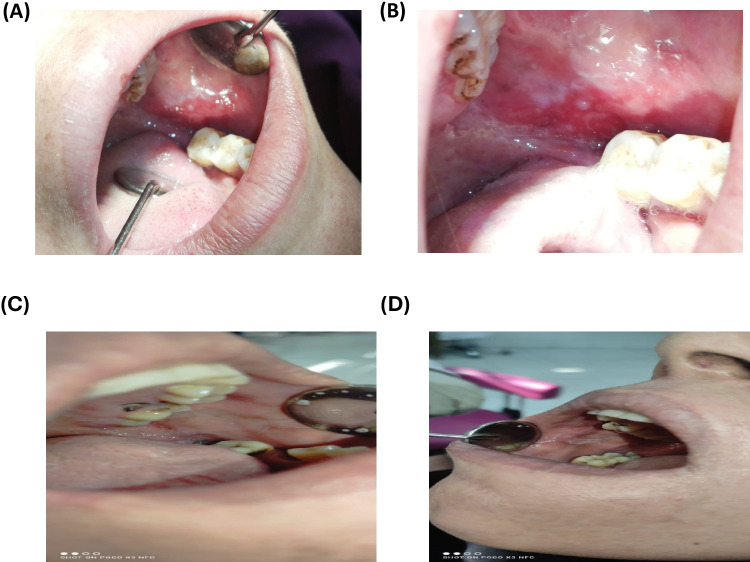
Photographs of (**A** & **B**) represent moderat erosion and ulceration in the buccal mucosa in lichen planus at baseline of the study group, Photographs of (**C** & **D**) represent healing of erosion and ulceration in the buccal mucosa of lichen planus after end of treatment of the study group.

**Fig. 7:**
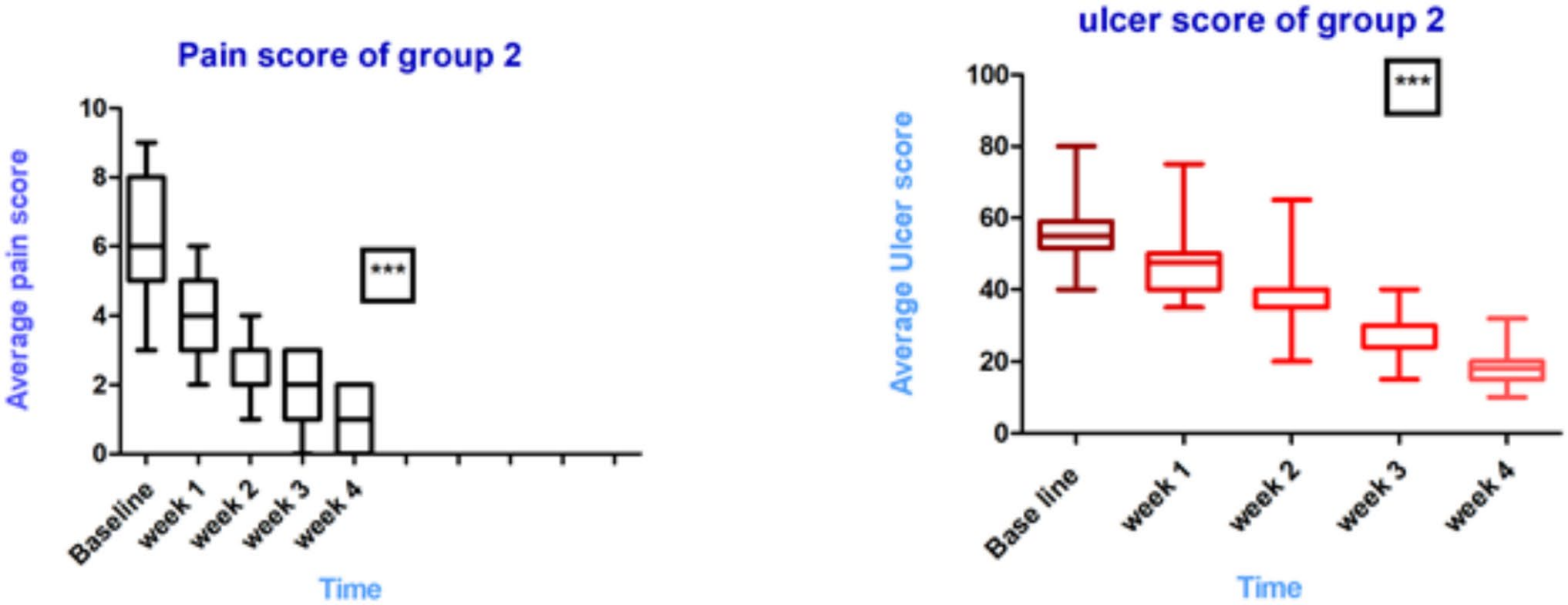
Kruskal-Wallis statistical test of the Pain score and the ulcer score of the patients in the study group (group 2). *** indicates highly significance

These results were in accordance with previous studies reporting that a 40–60 mg dose of corticosteroids is considered the gold standard dose in the treatment of OLP [110–113]. However, statistical analyses at the end of weeks two, three and four showed no significant differences between the two groups in terms of pain and ulcer scores confirming the efficacy of statins as adjuvant therapy. Moreover, the visual representation underscored the effectiveness of the intervention, aligning with the expected outcomes of improved mucosal health. Detailed data are provided in Table 6. As illustrated in Fig. 6, the buccal mucosa retrieved its characteristic normal pink color along with Wickham's striae and the absence of erosions, indicating a favorable response following treatment in the study group. These findings not only enhance our understanding of treatment efficacy but also highlight the importance of visual assessments in clinical practice, as they may correlate with improved patient comfort and quality of life. Notably, while statistical analyses at the end of Weeks 2, 3, and 4 did not demonstrate significant differences in pain and ulcer scores between the two groups, the observed normalization of mucosal appearance suggests a potential trend that warrants further exploration. For further assessment of the treatment’s effectiveness, the Kruskal–Wallis test was conducted to evaluate the significance of pain reduction between baseline and after four weeks of treatment, specifically for the study group. This analysis provided robust evidence supporting the effectiveness of the treatment, indicating a significant reduction (*P*-value < 0.0001) in pain and ulcer scores, as illustrated in Fig. 7. These results indicated the efficacy of the combination of a low dose of corticosteroids and topical application of ATV-Hyalugel in severe OLP. These results may be attributed to the ability of statins to accelerate epithelialization and wound closure by inhibiting leukocyte adhesion and extravasation at the inflammation site. This process reduces T-cell co-stimulation and inflammatory cytokine levels, facilitating early-stage wound repair. Additionally, statins enhance macrophage infiltration, which promotes fibroblast, keratinocyte, and endothelial cell proliferation. Furthermore, statins stimulate angiogenesis, encouraging macrophage infiltration and inducing vascular endothelial growth factor (VEGF) production and re-epithelialization [114–116]. In this context, HA serves as a key component of the formulated nanosystem, offering beneficial properties as a valuable adjuvant in treating inflammatory conditions, particularly in painful erosive lesions of the upper aerodigestive tract [117]. This is due to its moisture retention property, which creates an optimal healing environment, and its ability to promote cell migration and proliferation. The topical application of HA has been shown to alleviate pain and improve quality of life for patients with oral lesions, proving to be a useful adjuvant in the treatment of severe OLP [117, 118] Bruckmann et al.[117] revealed that topical administration of HA could play a role in the treatment of patients with inflammatory diseases via tissue repair and wound healing. Yildirim et al.[119] discussed the effect of topically applied HA on pain and palatal epithelial wound healing and found that HA demonstrated better complete epithelization. Combining the features of these two agents in a formulated nanosystem along with the hydrogel's physical properties presents significant advantages such as targeted delivery and controlled release, that maximize the therapeutic effects of statins at the pain site while minimizing systemic exposure. This combination can lead to improved healing outcomes and enhanced patient comfort.

**Table 6:** Comparison of the follow-up data of the two groups using Mann-Whitney test

**Week**	**Outcomes**	**Control group**	**Study group**	*P*-value
1	Pain	3 (2,4)	4(3,5)	0.0013*
	Ulcer score	40(37.5,50)	47.5 (40,50)	0.0063*
2	Pain	2 (2,3)	3(2,3)	0.3556
	Ulcer score	35 (30,39.5)	35 (35,40)	0.1340
3	Pain	2(1,3)	2(1,3)	0.8924
	Ulcer score	24 (23.5,32.5)	24(24,30)	0.5994
4	Pain	1 (0.25, 2)	1 (0,2)	0.6507
	Ulcer score	19 (15.25,20)	18(15,20)	0.5606

The current study demonstrated that topical ATV-hyalugel, used as an adjunct therapy, proved to be as effective as the control group in treating OLP, and it was well-tolerated. Our findings demonstrated the potential of topical ATV-Hyaulgel as a steroid-sparing adjuvant to significantly reduce systemic corticosteroid exposure while maintaining therapeutic efficacy in refractory OLP. This targeted approach offers a safer alternative to prolonged immunosuppressive therapies, addressing both clinical needs and patient safety concerns.

#### Limitations of the study

The limitations in this study include a small sample size, which may affect the broader applicability of our findings. Additionally, since ATV was employed as an adjunctive therapy for OLP, further investigations should assess the therapeutic potential of alternative topical statins, as well as their efficacy as monotherapy to gain a more comprehensive understanding of their impact on clinical outcomes.

#### Challenges

The potential association between statins and the onset of lichen planus remains controversial, primarily due to the predominance of case reports, which are considered limited in clinical evidence due to their anecdotal nature and lack of generalizability [120]. Patients may develop lichenoid eruptions shortly after initiating statin therapy or experience a delayed onset, which can range from a few weeks to several months or even years. This variability renders the determination of causality challenging and suggests that occurrences might be coincidental or influenced by factors such as genetic predisposition, concurrent medications, or underlying conditions [120]. These previous studies have explored the potential link between statin use and lichenoid eruptions, these studies have primarily involved systemic statin therapy using oral dosage forms [120]. In contrast, this randomized controlled trial offers a contrasting perspective that uniquely investigated the effects of statins in the oral cavity, utilizing a topical gel formulation, demonstrating that statin therapy significantly improves lichen planus symptoms, thereby highlighting their therapeutic potential rather than a causative role. This distinction is crucial, as topical application within the oral cavity may significantly differ from systemic administration in terms of absorption, efficacy, and side effects. These findings aimed to assess the therapeutic potential of a statin gel in managing lichen planus as adjuvant therapy to systemic corticosteroid, providing novel insights into the localized effects of statins in the oral environment. By focusing on a topical gel formulation, our study offers a fresh perspective on the role of statins in treating oral conditions, diverging from the systemic approaches reported in previous case reports. Further research, particularly well-designed clinical trials focusing on the long-term effects and potential therapeutic applications of statins in lichen planus, is necessary to clarify the relationship between these drugs and the condition. While our findings demonstrate ATV-Hyalugel’s therapeutic promise, key questions remain unresolved. Future studies must: Establish causality mechanisms through longitudinal pharmacovigilance studies tracking statin-naive OLP patients initiating topical ATV therapy, develop predictive biomarkers (e.g., HLA genotyping) to identify patients at risk for paradoxical lichenoid reactions, optimize regional drug delivery kinetics via intraoral microneedle patches to prevent systemic absorption, and Validate real world efficacy through decentralized trials in diverse populations using patient reported outcome measures for pain-free function. Crucially, this roadmap will definitively resolve the statin-OLP paradox while advancing equitable access to precision topical therapies. We will pioneer a global registry to prospectively track topical statin outcomes across genetic subpopulations, generating the first causal evidence specific to mucosal delivery.

#### Clinical translation considerations

Despite the promising clinical efficacy of ATV-Hyalugel, real-world translation requires addressing key barriers like (i) Cost implications of hyaluronic acid may be offset by 50% corticosteroid reduction and complication avoidance, (ii) Regulatory pathways will require GMP-compliant scale-up, where our pilot batches show excellent consistency, (iii) Initial adoption should target oral medicine centers with specialist training, as community dental implementation faces viscosity-application technique challenges, (iv) Accelerated translation could leverage chitosan's GRAS status and repurposed atorvastatin safety data for Phase III trial design optimization.

## Conclusion

The ATV-Hyalugel stands at the forefront of innovative therapies for OLP, heralding a transformative approach to patient care. This study showcases several pivotal advancements: the development of ATV-HAs via the thin film hydration method, yielding high entrapment efficiency (94.3%); comprehensive physicochemical characterization—including optimal particle size (221.2 nm) and zeta potential (–31.6 mV) ensuring formulation stability and efficacy; and a randomized clinical trial involving 90 patients that demonstrated significant improvements in pain and ulcer scores, with the ATV-Hyalugel group showing marked pain reduction from baseline after four weeks. Furthermore, the gel’s compatibility with low-dose corticosteroids enhanced therapeutic outcomes while preserving patient tolerability. This work bridges a critical translational gap in OLP management, where existing literature often siloes material science, clinical research, and regulatory strategy. We provide the first integrated solution from statistically optimized hyalurosomes to a randomized clinical trial demonstrating corticosteroid de-escalation (20 mg vs. 40 mg) enabling tangible reduction of high-risk systemic therapies. This pioneering therapy not only alleviates the burdens of chronic inflammation but also empowers patients to reclaim their quality of life, positioning ATV-Hyalugel as a cornerstone in the future of OLP therapeutic strategies.

### Future perspective

Looking ahead, advancing our understanding of the pathological mechanisms and risk factors underlying OLP remains a priority. Ongoing molecular and immunological research may unlock opportunities for more targeted and personalized therapies. Long-term clinical trials are warranted to validate the safety, optimal dosing, and sustained efficacy of ATV-Hyalugel, particularly in severe and recurrent cases of OLP. To enhance patient outcomes, future efforts will also focus on developing standardized diagnostic and management protocols, facilitating earlier intervention and improving quality of life. From a translational standpoint, we aim to scale this innovation globally through adaptable delivery formats such as lyophilized kits, making the formulation accessible and practical for real-world use. Ultimately, our patient-centered vision seeks to transform clinical success into everyday healing to restore comfort, confidence, and quality of life, from shared meals to unburdened smiles.

## Data Availability

Available upon request.
